# Investigating the Role of Auditory and Tactile Modalities in Violin Quality Evaluation

**DOI:** 10.1371/journal.pone.0112552

**Published:** 2014-12-04

**Authors:** Indiana Wollman, Claudia Fritz, Jacques Poitevineau, Stephen McAdams

**Affiliations:** 1 Sorbonne Universités, UPMC University Paris 06/CNRS, UMR 7190, Institut Jean le Rond, d'Alembert, F-75015, Paris, France; 2 Schulich School of Music, McGill University, 555 Sherbrooke Street West, Montreal, Quebec, H3A 1E3, Canada; Zhejiang Key Laborotory for Research in Assesment of Cognitive Impairments, China

## Abstract

The role of auditory and tactile modalities involved in violin playing and evaluation was investigated in an experiment employing a blind violin evaluation task under different conditions: i) normal playing conditions, ii) playing with auditory masking, and iii) playing with vibrotactile masking. Under each condition, 20 violinists evaluated five violins according to criteria related to violin playing and sound characteristics and rated their overall quality and relative preference. Results show that both auditory and vibrotactile feedback are important in the violinists’ evaluations but that their relative importance depends on the violinist, the violin and the type of evaluation (different criteria ratings or preference). In this way, the overall quality ratings were found to be accurately predicted by the rating criteria, which also proved to be perceptually relevant to violinists, but were poorly correlated with the preference ratings; this suggests that the two types of ratings (overall quality vs preference) may stem from different decision-making strategies. Furthermore, the experimental design confirmed that violinists agree more on the importance of criteria in their overall evaluation than on their actual ratings for different violins. In particular, greater agreement was found on the importance of criteria related to the sound of the violin. Nevertheless, this study reveals that there are fundamental differences in the way players interpret and evaluate each criterion, which may explain why correlating physical properties with perceptual properties has been challenging so far in the field of musical acoustics.

## Introduction

Music performance, in general, is a multimodal experience, involving perceptual, cognitive and motor components. When playing music, one of the most prominent sources of sensory information that accompanies audition is touch. As in the performance of many musical instruments, violinists are in intimate contact with the violin, the instrument being held very close to the player: the chin and the shoulder hold the violin body, the left hand the neck, and the right hand the bow. In addition to the sound, by fingering and drawing their bow across the strings, violinists receive vibrotactile feedback from their instrument as an implicit complement of the music produced. The tactile interaction between violinist and violin is thus inherent in the musical performance process and is probably related in one way or another to the feel of the violin. As reported by Marshall [1], “to be accepted by an artist, an instrument must not only sound correct but it must also feel correct”. Violinists thus probably use this tactile sensory input to perform better, to evaluate their own playing, and even to evaluate the quality of the instrument they are playing or are considering acquiring. In that sense, musical instrument evaluation is a complex perceptual, motor and cognitive task as well.

Previous work on the rating of violin quality has mainly focused on the characterization of the instrument itself, based either on comparative measurements of the violins' physical properties (e.g., [2]–[4]) or on evaluations of violin sound through listening tests [5], [6]. These two approaches have limitations as they only use a restricted set of audition-based criteria for the evaluation and neglect the player-instrument interaction. However, recently, Fritz, Curtin, Poitevineau, Morrel-Samuel and Tao [7] and Saitis, Giordano, Fritz and Scavone [8] have investigated violinists' evaluations while playing, bringing into consideration the sound and the “feel” of the instrument. The feel of the instrument is one aspect of violin playing and evaluation that has received little attention so far. Although many violinists report being really concerned about the feel of their instrument, there is a lack of consideration in the violin literature among authors who invoke this notion regarding what actually gives rise to the feel.

Marshall [1] stated that the vibrational modes of violin necks principally determine the feel of the instrument in that they exist at low frequencies to which the human skin is sensitive [0–1000 Hz]. According to him [1], [9], the detection of vibrations by the left hand of violinists is the basis for the sensation of the feel of the violin, sometimes referred to as its degree of “liveliness”. Hutchins [10] and Woodhouse [11] developed this idea by investigating how frequency matching between modes of the violin body and violin neck can enhance the sensation of “good feel”. In the violin literature, the notion of feel is thus commonly associated with vibrotactile feedback.

However, all these studies intended to quantify the characteristics of feel from vibrational measurements, but lack perceptual validation of their claims. A study of feel in a perceptual experiment is thus needed to quantify the extent to which the tactile modality alone can contribute to the perception of the overall quality of a violin. Eventually, this examination could help better correlate acoustical properties of the instrument with perceptual characterization of violins by deepening knowledge of the vibrational modes that influence the feel of violins more than of what determines the tonal quality of the instrument. Thus an initial investigation of how important the tactile sense is in violin playing and evaluation is required, particularly in relation to the contribution of the auditory modality.

### The role of auditory and tactile feedback in musical instrument performance

Several studies have investigated the role of one or more sensory modalities in musical performance. In particular, auditory and tactile modalities are regarded as important feedback mechanisms that enable musicians to control and experience their playing.

#### Auditory feedback in music

Since the paper of Lashley [12], research on musical instrument performance has demonstrated that an absence of auditory feedback does not impair or disrupt performance [13]–[15] and only slightly affects expressive performance [16]. Disruption can appear when auditory feedback content is altered, demonstrating the necessity of congruence between perception and action in music performance (see [17] for a review of the effects of altered auditory feedback). However, these studies have only dealt with musical performance on electronic keyboards, because it is easy to remove the auditory feedback with them. With regards to traditional musical instruments, Galembo [18] conducted a study with pianists who were blindfolded and “deafened” by way of headphones with white noise and showed that musician experts could discriminate the quality between three pianos primarily based on their feeling of the mechanical response of the instrument. This research needs to be extended to the performance of other musical instruments. Fulford, Ginsborg and Goldbart [19] recently explored the possible impairment in string ensemble playing by attenuating the auditory feedback to four violinists. Again, no serious effect of auditory attenuation was found on performers’ ability to play in a group, although the auditory masking was not complete.

Several authors have argued that players rely on their internal auditory representation of music, even in the absence of any audible sound [13], [20], [21]. They support the view that this “auditory imagery” would then guide their performance. Besides, as [15] points out, in the absence of auditory feedback players can still rely on tactile and proprioceptive information.

#### Tactile feedback in music

Two types of touch need to be distinguished. Active touch “refers to the combination of cues provided by tactile and kinesthesic receptors during active manipulation of objects in the environment. When stimuli are presented to a stationary observer, the cues arising from tactile receptors are referred to as passive touch” [22]. It is interesting to note that active touch and passive touch are inherently involved in violin playing: active touch mainly because of active bowing and fingering and passive touch mainly because of vibrotactile feedback during playing.

Several authors (e.g. [23], [24]) have reported that the tactile and kinesthetic senses are more important than passive touch in expert performance. Askenfelt and Jansson [25] proved that vibrations of many instruments including violins can be felt by the player. They suggested that this vibrotactile feedback plays an important role in ensemble playing (where hearing one’s own auditory feedback is not always possible) and in timing. Goebl and Palmer [26] extended this finding by highlighting the role of tactile feedback in performance timing accuracy for pianists. But, as Baader, Kazennikov and Wiesendanger [27] reported, information concerning the role of tactile cues in traditional musical performance and instrument evaluation is still scarce in the literature. Nevertheless, in the digital musical instrument literature, many researchers have shown the benefits of providing vibrotactile feedback to performers of computer-based bowed-string instruments so that the feel of traditional instruments can be mimicked, especially in the complex interaction characterizing the bowing action [28]–[30].

### Aim and research questions

The present paper explores the respective role of auditory and tactile (left hand) modalities involved in violin playing and evaluation. Since visual cues such as the color of varnish or the type of wood can influence musicians’ evaluations of instrument quality, and therefore constitute important cues for the recognition of violins, the current study was conducted under near-blind conditions.

As a first step, the goal of this project was to investigate the respective contribution of two sensory modalities to the evaluation of the sound and the feel of a violin by exploring how much auditory masking and tactile masking, respectively, affect the violinists’ evaluation of violin quality. Indeed, sensory masking is a way to uncouple the two modalities and so to study each sensory modality separately: with auditory feedback removal, tactile feedback is still available to the player and similarly, with tactile feedback deprivation, auditory feedback is unaltered. Can violinists evaluate violins without one or the other of two sources of sensory feedback, auditory and vibrotactile? Anecdotal evidence suggests that auditory feedback is of crucial importance in the assessment of violin quality – as audition seems to be the most important sensory modality in music performance – and is the most important source of sensory feedback for violin players in an evaluation process. Thus, although previous studies have reported that players are not much impaired by the removal of auditory information when performing, it remains an open question whether, and to what extent, auditory masking would be disruptive in quality rating and preference ranking tasks compared to vibrotactile masking.

On the other hand, one main aim in musical acoustics is to objectify the quality of an instrument by searching for mechanical correlates to perceptual attributes. Therefore, during quality evaluation tasks, while players would usually assess violins in terms of preference (hedonic approach), the use of different perceptual criteria (analytical approach) is often imposed by acousticians. This is indeed believed to lead more easily to mechanical correlates, even if the definitions of the criteria may be problematic in and of themselves and between-individual agreement is rarely studied. Our experimental protocol was thus designed to investigate how the two approaches (preference and different criteria ratings) relate to each other. The aim was also to assess how a list of criteria related to common specific attributes of violin playing and sound characteristics depend on each sensory modality, what their relative significance for professional violinists is and to what extent violinists agree when using them to evaluate violins.

To explore these issues, it is necessary to investigate first the extent to which auditory and tactile feedback can be eliminated without impairing violinists’ playing. The first part of the paper addresses this methodological issue. In the second part of the paper, we address the different questions reported above by testing the effects of playing conditions with different sources of sensory feedback on the answers given by violinists to a set of questions related to quality evaluation and to preference rating.

## Methods

### Participants

Twenty classical professional or semi-professional violinists took part in the experiment: eleven females and nine males with a mean age of 28 years (range: 18–54). They had all achieved a high level of expertise in violin playing, and each had at least 14 years of violin experience. None of them reported having auditory or tactile deficits. The violinists were paid for their participation.

### Ethics statement

**Figure 1:**
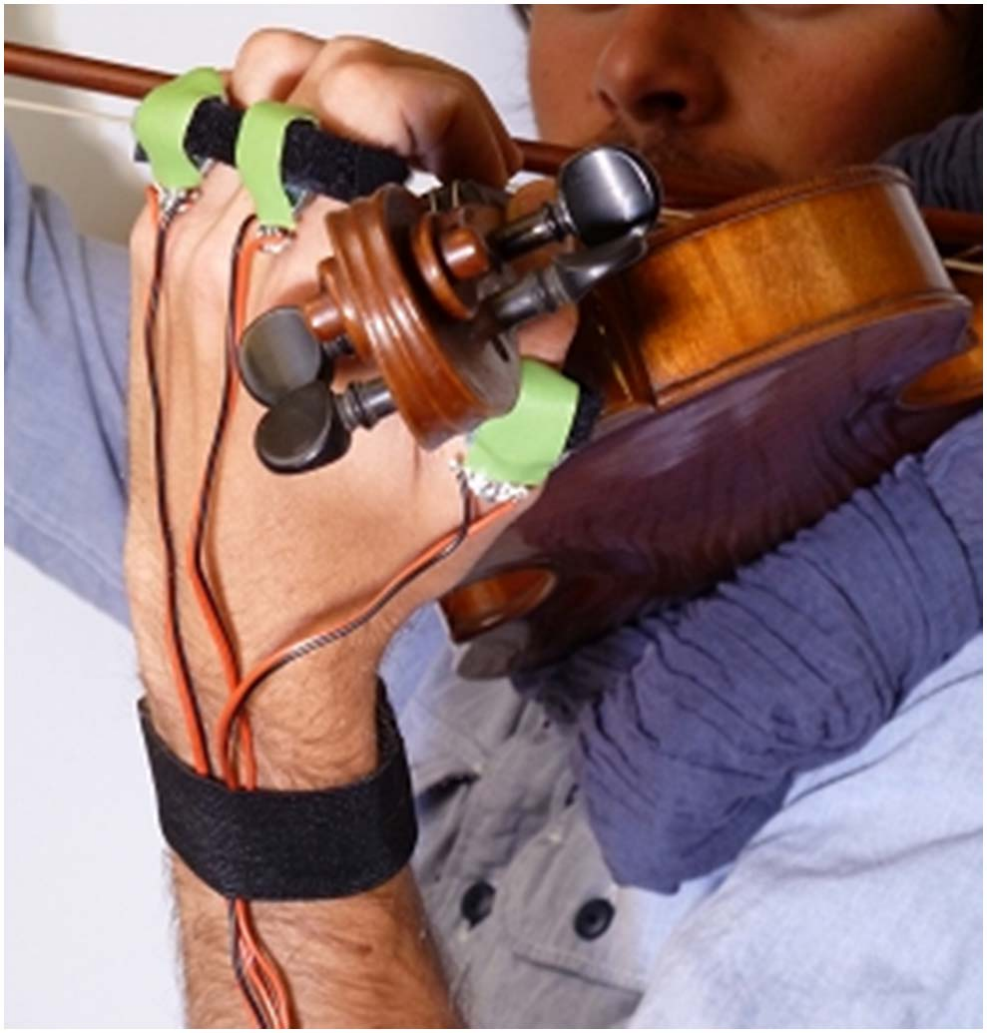
Vibrating rings worn by the violinists during the experiment.

This research was conducted according to the principles expressed in the Declaration of Helsinki. All participants provided written consent prior to participating in the experiment. This study has been approved by the McGill Research Ethics Board II (certificate 67–0905). The individual appearing on Fig. 1. of this manuscript has given written informed consent (as outlined in the PLOS consent form) to publish these case details.

### Violins

A set of five violins of different make and age, ranging from $20,000 to $30,000 and made between the early 18th and early 21st centuries, was assembled for this study. The violins were chosen by a luthier for their different playing and sound characteristics. Also, because we wanted violinists to evaluate violins according to the selected criteria only (see below), the five instruments were chosen to be as equal and standard as possible in size so that no violin could be clearly differentiated from the others on this basis. In the same way, identical shoulder rests (Kun Original model) were used for all five violins. The five violins will be referred to as VA,VB,VC,VD, and VE. Thus, the violin is treated as a fixed effect in our experimental design. Consequently, all the conclusions drawn here are restricted to this particular set of five violins. As in [8] experiment, each participant performed with her/his personal bow, which through constant use is considered to have become an extension of their bow arm. Moreover, this is how it works in real life: when trying out instruments, violinists typically use their own bows. We are aware that the bow can change the quality perceived, but that point is beyond the scope of this paper. We are indeed not interested in the quality of the violins as evaluated by players per se, but in the difference in quality evaluation by players in different conditions. What is thus important is to have the same player, using the same bow, evaluating the five instruments in each condition.

### Procedure

The experiment employed a blind evaluation task in which the violinists played and rated violins based on criteria related to violin attributes and according to their overall preference. The experimental session was divided into three playing conditions with different combinations of sensory feedback. Each violinist participated in all three conditions, and was free to play whatever music he or she liked. The experiment took place in a room specifically designed for musical performance and recording at the Centre for Interdisciplinary Research in Music Media and Technology at McGill University (floor surface = 26.7 m^2^, volume = 90.7 m^3^).

The order of the three conditions was counterbalanced across participants. They were asked to play and evaluate the set of violins, under normal playing conditions (N), with auditory masking (noA) and with vibrotactile masking (noT). There were no time constraints.

Within each playing condition, the order of presentation of violins was randomized and the participants were instructed to complete a series of evaluation tasks for one violin at a time. All answers were entered directly into a Matlab interface designed for the experiment by means of on-screen sliders. The evaluation procedure involved three main components:

1. For each violin, participants were first presented with eight different perceptual descriptors of violin playing and sound characteristics (see below), appearing one at a time in random order. They were asked to assess the magnitude, relevance and importance of each criterion, which was presented in the form “the violin is X”. The magnitude of each criterion was assessed by moving an on-screen slider along a continuous scale labeled “strongly disagree” on the left and “totally agree” on the right. They then had to answer the question: “Is this criterion relevant to you?” and explain their choice. If “yes”, then they had to assess the importance of the criterion in their evaluation of the violin by moving an on-screen slider along a “not important”/“very important” scale. If “no”, then they could indicate the reasons by ticking as many boxes as appropriate, namely, “I cannot evaluate this criterion on *this violin*”, “I cannot evaluate this criterion in this *playing condition*”, “I cannot evaluate this criterion because it *does not mean anything* to me”.
2. Then, ratings of the overall quality of each violin were made by moving an on-screen slider along a “bad quality”/“good quality” scale (coded 0–1).
3. Finally, when the first two components were completed for all five violins, participants were asked to rank the five violins by preference and were able to replay all of them. Equal ranking between violins was permitted. Preference ranking was performed by moving five on-screen sliders, whose colors represented the different violins, along the same “least-preferred”/“most preferred” scale. The violinists were instructed to use to entire slider range in their ranking, placing the most preferred and least preferred violins at the extremes of the scale and positioning the others in terms of relative preference along the scale. The positions of the five sliders along the preference axis thus constitute scores between 0 and 1 regarding violin preference.

Obviously, to be able to rate the different violins according to the different criteria, violinists needed to know the range covered by the five violins. Thus, violinists were given 20 min to play and freely explore the five violins under normal conditions to familiarize themselves with the violins at the beginning of the session. During this time, they were told they should get an approximate idea of the range of variation along each of the criteria within the set of violins. Also, participants had 10 min before each condition to familiarize themselves with the experimental playing situation by performing on their own violin taken as a reference.

No constraints were imposed on the musical style and repertoire used by the violinists during the experiment. Upon completing the session, participants filled out a questionnaire concerning their musical practice and details about the violin they own or play on regularly, including the maker's name, the year, and the origin. The experiment lasted approximately two and a half hours.

#### Evaluation criteria

The selection of criteria used in the experiment was based on previous studies investigating violin quality [7], [8]. The criteria were assumed a priori to be evaluatable through one or both sensory modalities under study, so that all criteria were presented under all sensory feedback conditions.

As in [8], we decided to present the criteria in the form of a short sentence followed by a short explanation to ensure a common interpretation of each descriptor. Because one purpose of this study was to test that the evaluation of the descriptor “Liveliness” relies upon tactile information only, no detailed explanation was given for this criterion in order to not orient violinists towards one particular modality. The eight criteria included:

EASE OF PLAYING: the violin is easy to play, speaks easily  =  the violin requires minimal effort to produce sound, easy to avoid wolf tones, easy to “get around” the instrument.

LIVELINESS: the violin is lively.

RESPONSIVENESS: the violin is responsive, speaks quickly  =  the violin has an immediate response to pressure and speed, there is no delay in response (almost no time between touching the string and the desired sound being produced).

SOUND RICHNESS: the violin has a rich and full sound  =  the violin produces a sound that is rich in harmonics and overtones.

DYNAMIC: the violin has a broad dynamic range, from piano to forte.

LOUDNESS/POWER: the violin is loud and powerful.

EVENNESS: the violin is well-balanced across the strings.

SOUND PALETTE: the violin has a broad sound palette with different colors.

### Apparatus – control of sensory loss

#### Visual feedback control

The experiment took place under low lighting conditions, and dark sunglasses were worn by the violinists to prevent detailed visual feedback. Indeed, running the experiment in a dark environment guarantees that participants are not able to visually recognize the instruments because of the color and texture of their wood and varnish. Nevertheless, they could see enough to comfortably play and complete the evaluation task on the computer screen.

#### Auditory feedback masking

To investigate the extent to which auditory feedback could be eliminated, a preliminary study was carried out. The combination of attenuating earmuffs in addition to in-ear monitors playing white noise was chosen to prevent the musician from hearing the airborne sound produced by the violin. We decided to use an auditory masking noise with a bandwidth of 20–20,000 Hz that has been shown to be effective in eliminating one’s own auditory feedback at all levels without exceeding tolerance levels [31].

The masking noise level for violin auditory feedback was determined in two steps. First, a volunteer violinist was recruited to record sound levels that typically exist in violin playing. He was equipped with head-microphones (Soundman OKM II Classic/Studio A3) and earmuffs (Bilsom Leightning L3, SNR 34 dB). He was asked to play forte on the G-string of a violin considered as the most powerful string. The sound level was recorded in the violinist’s ear canals after a first attenuation from the earmuffs. Unsurprisingly, it was found that the earmuffs did not sufficiently attenuate the sound level to make it inaudible and that the sound level measured at the left ear was stronger than the one at the right ear, due to the side on which the violin is held. The level of the masking noise was chosen to correspond to 6 dB above the violin sound level recorded at the left ear. In addition, due to their design, the in-ear monitors used to play the masking noise contributed to further reduce the sound level. As a second step, the masking noise level was calibrated using a B&K Sound Level Calibrator (type 4231) placed at a distance of 2 cm from the left earphone through a tube mimicking the external ear canal. In the end, it was found that the combination of earmuffs and in-ear monitors playing white noise at 90 dB SPL masked the violinists’ auditory feedback. Finally, passive anti-vibration material (Vibra Block Sound Deadening Material) was added to the chin rest to damp the sound that could be perceived via bone conduction through the jaw.

During the experiment, participants reported not being able to recognize the pitch of the notes being played, but were able to slightly detect that they were playing, ensuring a minimum playing comfort. One can infer that this feeling probably relates to bone-conducted feedback at the jaw and shoulder. However, it was impossible to eliminate completely the “sound” transmitted through bones for several reasons. One solution would be to add a huge and massive block that cut the vibrations at the chin and shoulder. But this could not be achieved without damping the violin itself. Another solution was to anaesthetize the body region of interest in each participant. Because the goal of the study was to let violinists play as comfortably as possible, and not only under auditory masking condition but under different playing conditions, this solution was not considered.

It should be noted that all violinists but one could evaluate violins under the auditory masking condition. Although the one exception had “naturally” performed on all five violins during the familiarization phase, she considered during the evaluation phase that no criterion was relevant to her because of the playing condition and thus decided to skip this particular condition.

#### Tactile feedback masking

As discussed previously, different contact points exist between the violinist’s body and the violin in a playing situation. Since the bow is the first mechanical link in the chain that produces sound, it is impossible to disturb the right hand holding the bow without impairing the playing. We thus decided to primarily mask the vibrations that can be felt by violinists through the left hand, which has been claimed to be an important cue to the perception of how a violin feels [1], [10], [11], and to simply attenuate the vibrations that can be perceived through the jaw and shoulder.

Three vibrating rings to be worn on the thumb, index and ring fingers of the violinist’s left hand were constructed (see Fig. 1). Each ring consisted of a small vibrator (Dayton Audio DAEX13 Mini Exciter Pair 13 mm) held tightly between the finger and an elastic band so that the vibrating surface was in direct contact with the skin of the finger. When vibrating, the rings transmitted the vibrations to the finger flesh and bone. It has been shown that skin sensitivity to vibratory stimuli is dependent on stimulus frequency in the range from near 0 Hz to approximately 1000 Hz [32]. The maximal sensitivity of the hand has been found to be in the region around 300 Hz [33]. The most sensitive frequency range for the skin of the hand is thus within the register of the violin, whose four open strings have fundamental frequencies at 196 Hz, 293 Hz, 440 Hz, and 659 Hz, the lowest note being G3 (196 Hz) [25]. We thus decided to use a tactile masking noise with a bandwidth of 10–1,000 Hz to feed the three vibrating rings.

To investigate whether and to what degree the vibrotactile feedback that reaches the left hand might be affected by tactile masking noise, a preliminary experiment was carried out prior to the present experiment. The goal was to determine approximately the masking threshold corresponding to the experimental set-up. Subsequently, a gain of 6 dBV (voltage relative to 1V) was added to this masking signal. Fourteen violinists with regular violin practice took part in the study. Participants were equipped with the three vibrating rings worn on the left hand at the beginning of the experiment and had to wear them during the whole session. However, the rings vibrated only during the tactile masking condition. Violinists were invited to play a scale at a dynamic of *forte* on their own violin and on a student violin available in the lab, in order to select two notes on each violin that they felt conveyed the strongest vibration to the left hand. The masking threshold was determined for each violin and for each note selected, employing a psychophysical adaptive staircase procedure. While continuously playing the selected note at a dynamic of *forte*, the 10–1,000 Hz vibrating stimulus was changed in level by the experimenter in successive, discrete steps, and the observer’s response to each stimulus presentation was either “violin felt” or “violin not felt”. The vibrating stimulus was initially too weak to mask the violin neck’s vibrations, so that the answer was “violin felt”; the level was then increased in steps until the violin neck’s vibration became imperceptible (ascending series). Five reversals in intensity (i.e., five turn-points corresponding to three “violin not felt” and two “violin felt” answers) were taken to estimate the masking threshold. Then, a sinusoidal signal was recorded as an RMS voltage value to calibrate the experimental set-up at the threshold level. The procedure was repeated twice for each note. There was little difference in the threshold level between two repetitions, but, unsurprisingly, the masking threshold was found to be dependent on the violin and the note played on it. The level of tactile masking noise was thus selected to correspond to the maximum masking threshold collected, converted to dBV units and then augmented by 6 dBV. In the end, the tactile masking noise level was set to 9.5 dBV in the rings, and vibrotactile attenuation at the jaw and shoulder was achieved by adding the same passive anti-vibration materials as described in the previous section to the chin and shoulder rests.

## Results

This section is divided into two parts, the first one (‘Effect of Sensory Masking’) addressing the first research question about the respective contribution of the two sensory modalities under study to violin evaluation, while the second one (‘How Violinists Evaluate Violins: Quality Criteria And Preference Evaluations’) deals with the second research question about the different approaches (perceptual criteria ratings, preference ratings) used to investigate the quality of a violin.

Following suggestions and recommendations by Wilkinson and Task Force on Statistical Inference [34], Bayesian statistical procedures were adopted in this study [35], using PAC software [36].

### Effect of sensory masking

Saitis et al. [8] showed that violinists are self-consistent in a preference ranking task under normal feedback playing conditions. For this reason, the normal condition is considered as the baseline situation in the following analyses. Therefore, in this section, the preference ratings and rankings made under the sensory masking playing conditions are discussed in relation to those made under the normal feedback playing condition.

#### Preference and overall quality ratings: within-participant consistency across sensory conditions

We use the Pearson correlation coefficient as an index of the impact of sensory masking on the preference ratings made in the normal condition. The mean Pearson correlation coefficient averaged over participants is 0.4 (SD = 0.4) between preference ratings made under N and noA and 0.5 (SD = 0.4) between N and noT. No greater correlation was thus clearly found between N vs. noA than between N vs. noT in preference ratings and the standard deviations are quite high and similar in both cases. The mean difference between N vs. noA and N vs. noT is –0.12, 95% CI [−0.37; 0.13], t(18) = –1.02, p = 0.16 (one-sided). Assuming an uninformative prior distribution (an uninformative prior expresses an initial “state of ignorance” about the parameter; no information is used other than what is contained in the data; no particular hypothesis is favored a priori, otherwise stated, uninformative means “Let the data speak for themselves.”), the Bayesian credibility interval indicates that given the data, there is a 84% probability that the true difference (between N vs. noA and N vs. noT) is lower than 0 (notated Pr*[true difference<0] = 0.84). The possibility of a positive (or null) population difference cannot be ruled out with sufficient confidence (we would state 0.95 as our minimal guarantee). Throughout the rest of the article, Bayesian statements will be denoted by Pr*; naturally they are conditional on the data at hand, but, for sake of brevity, the conditional notation is omitted.

This means that there seems to be no overall trend across participants as regards the levels of correlation between preference ratings made under the normal condition and each sensory masking condition (participants having a strong correlation between N and noT are not necessarily the same as the ones with a high correlation between N and noA). Hence, on average, auditory masking did not appear more disruptive than tactile masking in the preference ranking task.

In addition, as for the preference ratings, the mean Pearson correlation coefficients were computed between overall quality ratings made under the normal condition and each sensory masking condition. The mean Pearson correlation coefficients are 0.1 (SD = 0.6) between the overall quality ratings made under N and noA and 0.1 (SD = 0.4) between N and noT. In this case too, no clear difference between the noA and the noT conditions is observed.

#### Comparison with self-consistency across repetitions of the same preference ranking task

It is interesting to compare our results with those of Saitis et al.’s experiment [8] in which 20 violinists performed two exact repetitions of the preference ranking task under normal feedback conditions. Their experimental design is rather similar to ours. With their findings, the mean of across-participant Spearman correlation coefficients was quite high: ρ-mean = 0.62 (SD = 0.36). In the present experiment, after converting the ratings into rankings, the mean Spearman rank correlations between rankings made under normal and each sensory masking condition (N vs. noA, 19 participants/N vs. noT, 20 participants) are both equal to 0.35 (SD = 0.39 and 0.41, respectively). If we compare Saitis et al.’s results to our N vs. noA results, on the one hand, and to our N vs. noT results, on the other hand, the differences between their results and ours are similar: mean difference = 0.27, 95% CI [0.02; 0.51], t(37 or 38)  = 2.21, p = 0.02 (one-sided), and there is a 71.1% probability Pr* that it is higher than 0.20, considered as a substantial difference value. This result suggests that, as could be expected, violinists tend to show less variation when repeating the ranking task twice under the same normal feedback condition than when repeating the task with sensory masking. It is therefore likely that the differences observed in the preference ratings and rankings of the sensory masking conditions regarding the normal playing condition result from the deprivation of either auditory or vibrotactile feedback or both sources of feedback and not from a lack of consistency in the musicians’ ratings.

#### Overall quality and criteria ratings: within-participant coherence

The participants had to rate the magnitude of the eight selected criteria and their importance in their evaluations. Note that each time a criterion was reported not to be relevant, the corresponding importance was set to zero.

For each condition, violin and participant, the weighted sum of criteria was calculated as the sum of the products of each criterion’s magnitude rating and its importance rating, divided by the total sum of those importance ratings. The weighted sum of the ratings is thus another way to give a violin a score for a given condition and participant. We then correlated the violin scores with the overall quality rating for each participant in each condition. The mean correlation coefficients averaged over participants are shown in Table 1, which reveals a moderate to strong association between the way participants rated the criteria and the way they rated the overall quality of the violins under each playing condition. The participants thus appear to be very coherent in their evaluation.

**Table 1:** Mean Pearson correlation (averaged over participants) between the weighted sum of the criteria ratings and the overall quality ratings for the five violins under the three playing conditions.

	Mean correlation coefficient	Standard error of the mean (sample size)	95% Confidence Interval
COND N	0.85	0.05 (20)	[0.73; 0.96]
COND noA	0.67	0.11 (17*)	[0.43; 0.91]
COND noT	0.89	0.04 (20)	[0.81; 0.97]

*Three participants had missing data for at least three violins in the noA condition (in addition to violinist #6, two violinists considered that none of the eight criteria were relevant to them when evaluating three and four violins, respectively), so that the correlation was not computed.

#### Effect of sensory masking on preference rating

**Figure 2:**
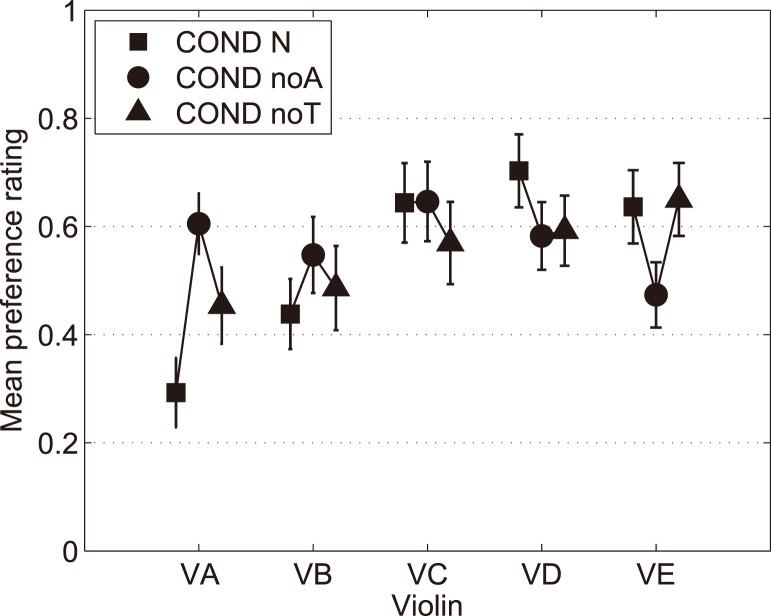
Mean preference ratings under the three playing conditions. The vertical bars represent the standard errors of the mean.

Figure 2 shows the mean preference ratings of the five violins under the three playing conditions.

Under conditions of sensory masking (circles and triangles in Fig. 2), the mean preference ratings are of relatively equal magnitude within the five violins (range noA: 0.47–0.65; range noT: 0.45–0.65), and are less different among themselves than in the normal reference condition (range N: 0.29–0.70). No violin stands out in the set of instruments. This means that there is less of a difference in preference among the instruments when eliminating not only auditory cues, but also, more interestingly, the vibrotactile information.

Figure 2 provides interesting new data on the role of auditory feedback. Under the auditory masking condition, the mean ratings of violins VA and VE fluctuate relative to the normal-feedback condition, whereas the ratings barely change for violin VC. Moreover, the direction of variation of the ratings between the normal and auditory masking conditions depends on the violin. For VA the mean rating is indeed higher when not heard, whereas the opposite happens for VE. From this observation, it can be hypothesized that there are three types of violins. Violins of the first type are preferred for their sound, becoming more ordinary, thus less preferred when they are not heard (i.e., “sound good” violins with no particular good feel). Violins of the second type are not preferred for their sound and are thus more preferred when not heard (i.e., “feel good” violins with no particular good sound). Lastly, some violins have something special that does not relate to their sound characteristics. These observations suggest that the contribution of audition to the evaluation of violins with respect to preference depends on the violin, and this is supported by statistical analyses, provided in Appendix S2.

In summary, this perceptual experiment demonstrated that violinists had sufficient cues to coherently evaluate violins under playing conditions with sensory masking. In particular the use of tactile cues for violin quality evaluation was revealed. Auditory masking was not more disruptive than tactile masking for preference and overall quality in general, but its effect depended on the violin. The experimental design also allows us to delve into the different methodological approaches used in violin quality evaluation. The issue is developed in the following section.

### How violinists evaluate violins: quality criteria and preference evaluations

#### Inter-participant variability

As a complement of Figure 2, Tables 2a and 2b show the number of times each violin was most preferred (first in the ranking) and least preferred (fifth in the ranking) by condition, respectively.

**Table 2:** Number of times each violin was most (a) or least (b) preferred by condition.

	Violin					
	VA	VB	VC	VD	VE	no answer
**A) Most preferred**						
COND N	1	1	7	6	5	0
COND noA	3	5	8	2	1	1
COND noT	2	4	5	3	6	0
**B) Least preferred**						
COND N	12	4	2	1	1	0
COND noA	2	4	5	3	5	1
COND noT	6	6	4	2	2	0

There is no clear consensus on the most preferred or least preferred violins in any of the three playing conditions. Under the normal playing condition, the violins can be divided into two groups. The first group would be violins VC, VD, VE, which were chosen about equally often, by between one quarter and one third of the participants (Table 2a). By contrast, violins VA and VB differentiate themselves as the least-preferred ones on average (Table 2b) and were preferred only once each (Table 2a). These violins constitute the second group. As can be seen from Figure 2, the variability of preference ratings across participants does not depend on the violin, because the bars representing the standard errors of the mean are of relatively equal magnitude across the five violins under all conditions. These results confirm that violin preference is highly individual [8], which orients further analysis towards within-participant analyses.

**Figure 3:**
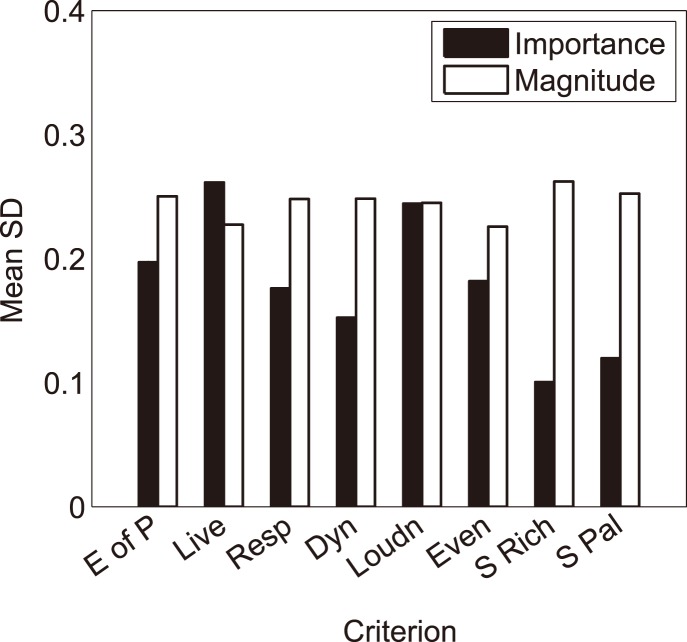
Arithmetic means over the five violins of the standard deviations of the importance and magnitude ratings, under normal playing conditions.

The fact that both magnitude and importance ratings were collected during the experiment enables us to test whether violinists agree on the importance of each selected attribute more than on the actual evaluation of those criteria in the normal feedback condition. This investigation intended to assess whether the inter-participant variability was smaller for the ratings of each criterion’s importance than for the ratings of each criterion’s magnitude. Figure 3 shows the arithmetic means over the five violins of the standard deviations of the importance and magnitude ratings.

As seen with the white bars on Figure 3, the mean standard deviations with regard to criteria magnitude are relatively equal among criteria (between 0.23 and 0.26). This suggests that, as expected, the large inter-participant variability does not exist in violin preference only but in the evaluation of specific attributes of violin playing and sound characteristics as well.

As seen with the black bars in Figure 3, the average standard deviations with regard to criterion importance vary a great deal across the rating criteria. Less inter-individual variability is observed for importance than for magnitude, because for all but two criteria, the average standard deviations of the importance ratings are lower than those of the magnitude ratings. At least for some criteria, violinists thus seem to agree more on their importance than on the magnitude of those criteria for different violins.

#### Overall quality vs Preference

Table 3 shows the mean correlations between preference ratings and overall quality ratings by condition, which reveals some interesting facts concerning the violinists’ ratings given the nature of the three playing conditions.

**Table 3:** Mean Pearson correlation (averaged over subjects, df = 3) between the preference ratings and the overall quality ratings for the five violins under the three playing conditions.

	Mean correlation coefficient	Standard error of the mean (sample size)	95% Confidence Interval
COND N	0.11	0.12 (20)	[–0.14; 0.36]
COND noA	0.12	0.13 (19)	[–0.15; 0.39]
COND noT	0.32	0.12 (20)	[0.07; 0.57]

Preference ratings are poorly or weakly correlated with the overall quality ratings in all of the conditions. In other words, one can give a strong, even the best, rating to a violin in an “objectivized” evaluation task – overall quality rating – and then poorly rate that same violin when it comes to preference. What is also remarkable is that the same phenomenon occurs under all conditions, suggesting that violin preference judgments are commonly unrelated to violin “value” judgments. Incidentally, multiple regression did not find any significant relationship between preference and the eight criteria.

#### Towards a hierarchy of criteria

**Figure 4:**
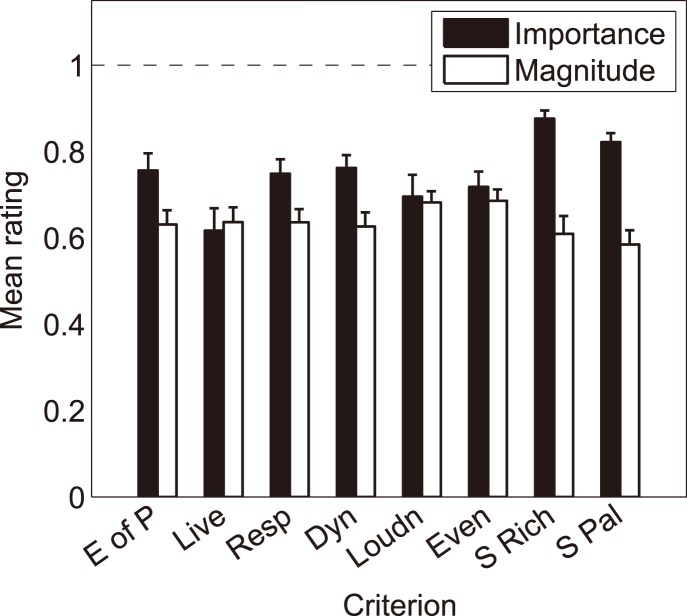
Means of the importance and magnitude ratings under normal playing conditions, averaged over the five violins. The vertical bars represent the standard errors.

Figure 4 shows the mean importance and magnitude ratings, averaged over the five violins. The data are plotted as a function of the eight criteria, and the error bars represent the standard errors.

Averaged over the violins, the means of the importance ratings are fairly high for all criteria, ranging from 0.62 (Liveliness) to 0.88 (Richness). The dependence of observed criterion importance on the violins is not strong; For each criterion the overall raw effect of violin is measured as the Root Mean Square (RMS) of the pairwise mean differences between violins, and the p’s ­ corresponding to a one-way repeated-measures ANOVA on the importance ratings ­ range from 0.17 to 0.99 for all criteria but Richness for which p<0.01. We can conclude that differences among violins are small for the whole population of participants and for all criteria, because the upper RMS limit (defined as Pr*[|population RMS| < RMS limit] = 0.95), obtained for Liveliness, is only 0.08 on a scale of [0–1]. (Although the effect of violins on the importance ratings of the “Sound Richness” criterion is significant, its RMS upper limit is only 0.07).

The means of the magnitude ratings over the violins range from 0.58 (Palette) to 0.69 (Evenness) (Fig. 4). Although the differences among the violins seem to be more pronounced than for the importance ratings, no significant effect of violin is found for any of the criteria (p’s – corresponding to a repeated-measures ANOVA on the magnitude ratings – ranging from 0.36 to 0.97). This results from the large inter-variability. Furthermore, as Pr*[|population RMS| <0.17]≥0.95 for all criteria and 0.17 is rather small, the violins can be considered as rather similar.

Note that both criteria directly related to the sound of the instrument, namely “Sound Richness” and “Sound Palette”, were most commonly rated as important in the violinists’ evaluations as expressed by their high mean importance rating (Fig. 4) and by low values of their standard deviations (Fig. 3), around 0.10. By contrast, the largest variabilities were found in the ratings of the importance of “Liveliness”, “Loudness”, “Evenness” and “Ease of Playing” (average standard deviations greater than 0.2 in Fig. 3). The fact that the variances are lower for importance than for magnitude is confirmed, using the Pitman-Morgan test for differences between correlated variances, for “Sound Richness”, “Sound Palette” and “Dynamics”: for the first two criteria, p≤0.02 for all violins, while for the third, p≤0.05 for all violins but VB for which p = 0.06. For the other criteria, there is no clear trend with variations from one violin to one another (p’s ranging from 0.01 to 0.64). These observations support the view that criteria related *a priori* more directly to violin sound are of larger importance in violinists’ perceptual evaluations, whereas violinists agree less on the importance of criteria related *a priori* to both violin playing and sound characteristics.

#### Perceptual foundation of the criteria

Because the instructions allowed violinists to decide whether a given criterion was relevant or not, the results contained many empty cells. Therefore, the data on criterion assessments were not subjected to inferential statistical analyses. The ranges of the number of times each criterion was found to be relevant to the violinists across violins are given in Table 4. The data are presented by criterion and condition. This classification being based on averaging the results over the 20 participants, it may thus be more complex at an individual level.

**Table 4:** Range of number of times each criterion was found to be relevant across violins in each condition.

	COND N	COND noA	COND noT
*Sample size*	*20*	*19*	*20*
Ease of Playing	[20–20]	[16–18]	[18–19]
Liveliness	[18–20]	[11–13]	[18–19]
Responsiveness	[20–20]	[15–17]	[19–20]
Dynamics	[20–20]	[10–12]	[20–20]
Loudness	[19–20]	[12–13]	[19–20]
Evenness	[20–20]	[11–13]	[19–20]
Sound Richness	[20–20]	[9–11]	[20–20]
Sound Palette	[20–20]	[6–9]	[20–20]

The reasons reported for judging a criterion irrelevant were attributed for 90.7% to “this playing condition”, 8.7% to “this violin”, and only 0.6% to “this criterion doesn't mean anything”. Hence, the list of selected criteria would seem to make sense to the violinists. This finding is coherent from another viewpoint with the previous observation that in all playing conditions the criterion assessments accurately predicted the overall quality ratings.

Under the normal conditions of feedback, almost all violinists evaluated almost all criteria, and all but one or two violinists found the criteria relevant with a loss of tactile sensation. Under the auditory masking condition, however, on average only half of the violinists assessed the criteria, the exact number depending on the criterion. The fact that a much greater number of violinists found some criteria more relevant in the tactile masking condition than in the auditory masking condition again suggests that, on average, an absence of auditory feedback affects the evaluation of these particular criteria more than does the absence of vibrotactile feedback. Nevertheless, considering that under the noA condition, all criteria were each still relevant to about one third of the violinists, one could argue that the auditory masking is not necessarily always more disruptive at an individual level.

The table provides information on the respective contribution of each sensory modality to the evaluation process of the criterion in question. The absence of auditory feedback has a strong effect on the relevance of “Sound Richness” and “Sound Palette” and a moderate effect on the relevance of “Ease of Playing”, and “Responsiveness”; in contrast, the absence of vibrotactile feedback primarily affects the relevance of those latter criteria and “Liveliness”, although the effect is slight. There is thus some evidence of three separate groups of criteria within the list, concerning the sensory modality to which each criterion is related. One group would consist of criteria mainly related to audition, namely “Sound Richness”, “Sound Palette”. A second group would consist of four criteria that relate to both auditory and tactile modalities. These are “Liveliness”, “Dynamics”, “Loudness/Power” and “Evenness”. Finally, the group consisting of “Responsiveness” and “Ease of Playing” would include criteria that depend to a large extent on tactile cues.

In summary, this perceptual experiment demonstrated that preference and overall quality ratings do not lead to the same evaluation, which calls into question the methodology normally used by acousticians when searching for acoustical correlates. Despite low agreement among players on the preference and on the magnitudes of the selected criteria, three groups of criteria could be identified on average and greater agreement on the importance of “Sound richness” and “Sound palette” in the evaluation was revealed.

## Discussion and Conclusions

The present paper has investigated the role of auditory and tactile feedback in the left hand in violin quality evaluation and preference rating. One question raised in the introduction was to know whether and to what extent violinists could play on and evaluate violins without feedback from one of these sensory systems. In this experiment, all 20 violinists were able to carry out the evaluation task under the condition with tactile masking in the left hand (noT) and all but one under the auditory masking condition (noA). These observations mean that violinists can play and evaluate violins without feeling violin vibrations in the left hand or without hearing themselves. So in both cases, they appear to have sufficient cues from the other sources of sensory information to overcome the impairment induced by masking to some extent. This study can be interpreted as tentative support for Finney’s (1997) observations that the absence of auditory feedback does not cause severe playing impairment (otherwise violinists would not have been able to carry out the task) and extends these findings to stringed instrument playing. It furthermore provides new data on the effect of vibrotactile masking on violin evaluation.

It was assumed that the more a given type of sensory masking was disruptive in the evaluation task compared to the normal feedback condition, the more this sensory modality would be important in violin playing and evaluation. As we cannot dissociate the change in individuals' response strategies and the change in the violinists’ actual ability to perceive/evaluate the violins, the term “disruptive” includes both factors.

Considering the preference ratings, comparison of our rank correlations for normal vs. auditory masking and normal vs. tactile masking with the repeated ratings in Saitis et al.’s experiment [8] shows greater variability in our case, suggesting that the sensory perturbation reduces correlation, but to a similar degree for auditory masking and tactile masking. Moreover, if on average across participants auditory masking seemed to have a greater effect on preference than did tactile masking (the mean preference ratings under normal conditions were closer to those under tactile masking than under auditory masking, Figure 2), this was not necessarily the case at an individual level because no greater correlation was consistently found between preference ratings under normal and tactile masking than between ratings under normal and auditory masking. These individual differences may result from a possible division of the violinists into two groups as regards their preference: Some violinists report that they would not choose a violin if they did not like its sound (those who rely on auditory cues more than on tactile cues), whereas other violinists claim that they do not care much about the sound (unless it is really too “bad”, for instance, too nasal), because they can usually “shape” the sound the way they want. In addition, the contribution of auditory feedback to violin preference depends on the violin. Indeed, auditory masking was found to affect some violin ratings positively, others negatively, and had no effect on one violin.

Considering the quality ratings, evaluation of the rating criteria appeared more difficult in the auditory masking condition compared to the tactile masking condition, i.e., the number of violinists who found some criteria relevant was much smaller in the auditory masking condition than in the tactile masking condition (see Table 4). However, although there is a strong asymmetry of the effect of sensory masking on the relevance of evaluation criteria (very slight effect for tactile masking, moderate to strong effect for auditory masking), this result cannot be easily generalized at the individual level because all criteria were still relevant to a third of the participants in the auditory masking condition.

One may wonder whether the perceptual judgments were completely random in the sensory masking condition or whether the players were still be able to make a judgment based on other available information. A list of evaluation criteria drawn from the literature and related to common specific attributes of violin playing and sound characteristics was chosen for the evaluation process. Participants were asked to assess the magnitude and importance of each criterion in the evaluation of each violin. Under all sensory feedback conditions, a moderate to strong correlation between criterion ratings and overall quality ratings was found, demonstrating an impressive coherence in the way violinists carried out the evaluation task. In both sensory masking conditions, it can thus be argued that violinists had sufficient cues from the other sources of sensory information to overcome the impairment induced by masking to some extent. So the inconsistency previously revealed for preference ratings across conditions can be attributed to sensory loss.

Incidentally, the weights of each criterion's contribution to the evaluation of the violin, estimated from the importance ratings, were shown to be of limited value by the fact that similar correlations were found with equal weights (see Appendix S1). Furthermore, rating the importance of each criterion in the evaluation is time consuming. As a consequence of these two findings, we would not recommend asking participants to rate criterion importance in future studies investigating instrument quality.

If the results of this experiment point out that tactile cues are used in violin evaluation, remember that in the auditory masking condition, the tactile information not only consisted of the vibrotactile feedback at the left hand, but also consisted of tactile feedback at the bow hand. Since there was no masking of the right hand, the haptic and tactile sensation from the bow certainly played an important role. The way the string is excited by the bow and how its vibration depends on the interaction between the bow, the string and the violin body, can most probably be perceived through the bow and thus may provide tactile cues that are essential for the evaluation of some criteria (for instance “Responsiveness” and “Ease of playing”). This point needs further investigation to better understand how violinists make use of the vibrotactile feedback in the right hand. To test a double, auditory and tactile masking condition could provide insight into the additive effect of the masking and could be a way to address this question. This was however not possible due to the duration of our experiment, and the associated risk of compounding issues of increased fatigue. In addition, although we can observe that something passes through the tactile sense because evaluation was not completely disrupted by auditory masking, we cannot quantify the extent to which that tactile information is used in real life. Therefore, it cannot be ruled out with certainty that participants changed their evaluation strategy in the sensory masking conditions compared to the normal condition considered as the baseline situation. For instance, we cannot entirely exclude the possibility that the masking equipment diverted violinists’ attention from the evaluation task. Nevertheless, considering the complexity of the task, it seems highly unlikely that participants were able to learn the task so quickly and to be so consistent in their ratings if they did not use tactile information in actual practice, even without being conscious of it.

Furthermore, the observation that the relative importance of the auditory and tactile information depends on the violinist does not seem to be attributable to the background of the participants. Although no constraints were imposed on the musical style and repertoire used during the experiment, it is interesting to note that all violinists played classical music, ranging from simple scales to short excerpts from the most famous violin concertos. The inter-individual differences thus cannot be explained by the type of music they played to evaluate the instruments. Moreover, responses to questionnaires on the music education of the participants do not provide information about the different strategies used in the experiment, because they indicate that all participants have the same background in music, play essentially classical music, and almost all of them play a second instrument. Given the strong inter-individual differences that are revealed in most studies of violin evaluation, future research could perhaps examine in greater detail, and on a larger population of violinists, whether any relation exists between aspects of musical training and violin evaluation.

As regards our second research question, surprisingly weak correlations were found between how violinists objectively assessed the overall quality of the violin and how they rated and ranked the violins in terms of preference, under any of the playing conditions. While a simple combination of criteria – assigning them equal weight – was found to be well correlated with the overall quality rating, no consistent association between preference and the eight criteria was found across violins. It thus seems that the two types of ratings are at best weakly related for the musicians at a psychological level. Preference would thus be based on criteria other than those used in this and other studies. The components of preference have not been identified at this stage. The pleasure of playing could be an important aspect of preference, but its inherent complexity may not easily lend itself to quantitative and analytic measures. This result could also be explained by the fact that decisions can be different depending on whether the violins are evaluated comparatively or separately. The violins were rated together on the same scale in the preference rating task, whereas they were rated in isolation in the quality rating task (with one scale for each violin), and this can lead to evaluation reversals (see [37]). Indeed, given that the first task was a more direct way of comparing the instruments, it may have emphasized small differences that are otherwise difficult to evaluate independently. In summary, the results of the present experiment questions the usual methodology used by acousticians and indicates that the type of rating – overall quality vs. preference – should be further investigated and carefully considered in designing an experiment that aims to study instrument evaluation by players.

The search for acoustical correlates would be easier if the most important criteria for players were known, independently of the violin. The criterion evaluation data show less inter-participant variability for ratings of criterion importance than for the magnitude ratings of each criterion. This result completes Saitis et al.’s [8] observations that violin quality evaluation probably differs between performers “not because different performers prefer violins with largely different qualities, but because the perceptual evaluation of violin attributes widely considered to be important for a good violin vary across individuals” (p. 4011). Our study also demonstrates that the importance of the criteria to the overall evaluation depends little on the violins played, suggesting that violinists have their own list of criteria ordered in terms of significance. Incidentally, our results show that in their criterion evaluations under normal feedback conditions, violinists agree on the importance of criteria directly related to violin *sound* (namely “Sound Richness” and “Sound Palette”) more than on those related to *playing characteristics*. In the first place, it can be argued that criteria based on auditory cues are taken as the major criteria used by violinists. The largest agreement on the importance of “Sound Richness” and “Sound Palette” gives us clues concerning the direction in which to search for acoustical correlates. However, this is hindered by the low agreement among players on the magnitude of these criteria, and indeed our experiment sheds light on the reason for such a poor agreement. Two potential reasons can indeed be proposed for a low between-individual agreement for the magnitude. First, violinists play with different techniques (bowing speed and force, position of the bow on the strings) and thus do not really evaluate the same “instrument” as a result of the interaction between the player and the physical instrument. Second, there are fundamental differences in the way players interpret and evaluate each criterion. While no conclusion can be drawn concerning the first source with our experiment, the fact that some players consider “Sound Richness” as a relevant criteria without sound show that their definition and perceptual evaluation of it are necessarily different from the players who absolutely need the sound to evaluate the instrument. This observation could suggest that these terms do not relate unequivocally to sound properties for all violinists, but could be based on multisensory information processing as well. If “Sound Richness” and “Sound Palette” can be evaluated without hearing the sound of the violin, how can acoustic signal properties alone – such as spectral centroid – account for the perceptual criterion ratings? This sheds light on why correlating physical properties with perceptual properties has been challenging so far. More studies like [38] are thus needed to tease apart the fundamental differences that exist in the way players interpret each rating criterion.

Our investigation intended as well to establish a correspondence between the different violin attributes and the sensory modality they may be associated with. Despite this large inter-variability, three separate groups of criteria could be identified that lead to directions that should be favored in the search for acoustical correlates. One group consisted of criteria associated primarily with the auditory modality (“Sound Richness” and “Sound Palette”), another group included criteria associated to a large extent with the tactile modality (“Responsiveness” and “Ease of Playing”), and a third group included criteria associated with both auditory and tactile modalities in a more balanced proportion (“Liveliness”, “Dynamics”, “Eveness”, “Loudness”).

Despite the fact that musicians and acousticians agree that the “feel” of traditional instruments – as opposed to their sound – is important, no study had been conducted so far to investigate how that feel is important for the evaluation of instruments. This study clearly shows that tactile information does play a role in instrument evaluation as violinists’ evaluations were not completely disrupted by auditory masking. It also points to the possible multisensory processes that are likely to occur during instrument evaluation and for which more data need to be collected.

## Supporting Information

Appendix S1 Overall quality and criteria ratings.
(DOCX)

Appendix S2 Effect of violin on preference ratings under noA condition.
(DOCX)

Dataset S1 Preference ratings. Results table of the 20 subjects (column 1) across the 3 sensory feedback conditions (N, noA, noT in column 2). The preference ratings of the 5 violins (order VA, VB, VC, VD, VE) are indicated in columns 3∶7.
(TXT)

Dataset S2 Criteria ratings and overall quality ratings. Results table of the 20 subjects (column 1) over the 3 sensory feedback conditions (N, noA, noT in column 2), and the 5 violins (column 3) across the 9 criteria including « overall quality » (column 4). The following columns (5∶11) correspond to the ratings of each criterion magnitude, relevance and importance (see section procedure). Column 12 is relevant only for the overall quality ratings.
(TXT)

## References

[1] Marshall KD (1985) Modal analysis of a violin. Journal of the Acoustical Society of America 77(2):695–709.

[2] Dünnwald H (1991) Deduction of objective quality parameters on old and new violins Journal of the Catgut Acoustical Society, 1–5.

[3] Bissinger G (2004) Contemporary generalized normal mode violin acoustics. Acta Acustica united with Acustica 90:590–599.

[4] Bissinger G (2008) Structural acoustics of good and bad violins. Journal of the Acoustical Society of America 124:1764–1773. 19045666 10.1121/1.2956478

[5] Willgoss R, Walker R (2007) Discernment of the sound of a violin. In Proceedings of the 8th World Scientific and Engineering Academy and Society International Conference on Acoustics and Music: Theory and Applications, Vancouver, Canada. 1–6.

[6] Fritz C, Woodhouse J, Cheng FP-H, Cross I, Blackwell AF, et al (2010) Perceptual studies of violin body damping and vibrato. Journal of the Acoustical Society of America 127(1):513–524. 20058996 10.1121/1.3266684

[7] Fritz C, Curtin J, Poitevineau J, Morrel-Samuels P, Tao F-C (2012) Players preferences among new and old violins. Proceedings of the National Academy of Sciences of the USA 109(3):760–763. 22215592 10.1073/pnas.1114999109 PMC3271912

[8] Saitis C, Giordano BL, Fritz C, Scavone GP (2012) Perceptual evaluation of violins: A quantitative analysis of preference judgments by experienced players. Journal of the Acoustical Society of America 132(6):4002–4012. 23231129 10.1121/1.4765081

[9] Marshall KD (1986) The musician and the vibrational behavior of a violin. Journal of the Catgut Acoustical Society 45:28–33.

[10] Hutchins CM (1985) Effect of an air-body coupling on the tone and playing qualities of violins, Journal of the Catgut Acoustical Society. 44:12–15.

[11] Woodhouse J (1998) The acoustics of “A0-B0 mode matching” in the violin. Acustica - Acta Acustica 84:947–956.

[12] Lashley K (1951) The problem of serial order in behavior. In L.A. Jeffress (Ed.), Cerebral mechanisms of behavior. New York: Wiley.

[13] Gates A, Bradshaw JL (1974) Effects of auditory feedback on musical performance task. Perception and Psychophysics 16:105–109.

[14] Banton LJ (1995) The role of visual and auditory feedback during sight-reading of music. Psychology of Music 23:3–16.

[15] Finney SA (1997) Auditory feedback and musical keyboard performance. Music Perception 15(2):153–174.

[16] Repp BH (1999) Effects of auditory feedback deprivation on expressive piano performance. Music Perception 16(4):409–438.

[17] Pfordresher PQ (2006) Coordination of perception and action in music performance. Advances in Cognitive Psychology 2:183–198.

[18] Galembo A (2001) Perception of musical instrument by performer and listener (with application to the piano). In Proceedings of the International Workshop on Human Supervision and Control in Engineering and Music, Kassel, Germany, 257–266.

[19] Fulford R, Ginsborg I, Goldbart J (2012) Functions and uses of auditory and visual feedback: exploring the possible effects of a hearing impairment on music performance. In Proceedings of the 12th ICMPC-ESCOM Conference. Thessaloniki, Greece, 335–343.

[20] Gordon E (1993) Learning sequences in music: Skill, content, and patterns. Chicago, IL: GIA Publications. (Original work published 1980).

[21] Keller PE, Appel M (2010) Individual differences, auditory imagery, and the coordination of body movements and sounds in musical ensembles. Music Perception 28(1):27–46.

[22] Weisenberger JM (2008) Cutaneous Perception. In Blackwell Handbook Of Sensation and Perception. Edited by E. Bruce Goldstein. Copyright © 2001, 2005 by Blackwell Publishing Ltd.

[23] Keele SW (1973) Attention and Human Performance. Goodyear Publishing Company.

[24] Hollerbach JM (1990) Planning of arm movements. In D. Osherson, S. Kosslyn, & J. Hollerbach, Eds., Visual cognition and action. Cambrige, MA: MIT Press, 213–242.

[25] Askenfelt A, Jansson EV (1992) Vibration Sensation in Stringed Instrument playing. Music Perception 9(3):311–350.

[26] Goebl W, Palmer C (2008) Tactile feedback and timing accuracy in piano performance. Experimental Brain Research 186:471–479. 18193412 10.1007/s00221-007-1252-1

[27] Baader AP, Kazennikov O, Wiesendanger M (2005) Coordination of bowing and fingering in violin playing. Cognitive Brain Research 23:436–443. 15820650 10.1016/j.cogbrainres.2004.11.008

[28] Nichols C (2002) The vBow: A virtual violin bow controller for mapping gesture to synthesis with haptic feedback. Organised Sound, 7, 215–220.

[29] Florens J-L (2004) Expressive bowing on a virtual string instrument In Gesture-based communication in human-computer interaction, 5th International Gesture Workshop, Eds. Camurri A & Volpe G, Springer, 2004, 487–496.

[30] Sinclair S, Wanderley M M, Hayward V, Scavone G (2011) Noise-free haptic interaction with a bowed-string acoustic model. In 2011 IEEE World Haptics Conference (WHC), 463–468.

[31] Shliesser HF, Coleman RO (1968) Effectiveness of certain procedures for alteration of auditory and oral sensation for speech. Perceptual and Motor Skills 26:275–281. 5642536 10.2466/pms.1968.26.1.275

[32] Verrillo RT (1962) Investigation of some parameters of the cutaneous threshold for vibration. Journal of the Acoustical Society of America, 34(11), 1768–1773.

[33] Verrillo RT (1992) Vibration sensation in humans. Music Perception 9(3):281–302.

[34] Wilkinson and Task Force on Statistical Inference, APA Board of Scientific Affairs (1999) Statistical Methods in Psychology Journals: Guidelines and Explanations. American Psychologist 54:594–604.

[35] Rouanet H, Bernard J-M, Bert M-C, Lecoutre B, Lecoutre M-P, et al. (2000) New Ways in Statistical Methodology: From Significance Tests to Bayesian Inference (2nd edition). Peter Lang, Bern, SW.

[36] Lecoutre B, Poitevineau J (1992) PAC (Programme d’Analyse des Comparaisons): Guide d’Utilisation et Manuel de Référence. CISIA-CERESTA, Montreuil, FR. Freely available http://www.univ-rouen.fr/LMRS/Persopage/Lecoutre/Eris.

[37] Hsee CK, Loewenstein GF, Blount S, Bazerman MH (1999) Preference reversals between joint and separate evaluations of options: A review and theoretical analysis, Psychological Bulletin. 125(5):576–590.

[38] Saitis C, Fritz C, Guastavino C, Scavone GP (2013) Conceptualization of violin quality by experienced performers. In Proceedings of the Stockholm Music Acoustics Conference 2013, SMAC 2013, Stockholm, Sweden. 123–128.

