# Supplementary material for: Investigating the Role of Auditory and Tactile Modalities in Violin Quality Evaluation
Source: PLoS One. 2014 Dec 4;9(12):e112552. doi: 10.1371/journal.pone.0112552 (PMC4256376; doi:10.1371/journal.pone.0112552)
Supplement: Appendix S1 — Overall quality and criteria ratings. (DOCX) [file pone.0112552.s001.docx]

**Appendix 1: Overall quality and criteria ratings**

Giving the same weight to all criteria of the list (i.e., calculating the sum of the importance ratings divided by the total number of relevant criteria) gives very similar correlation coefficients, although they are slightly smaller. The error in estimation of a participant is measured as the absolute difference between his/her overall quality rating and the estimated quality (whether weighted or unweighted) averaged over the five violins. It is interesting to see whether the weighting reduces this error of estimation. The differences between the error with weighting and the error without are rather similar in N, noA and noT conditions: mean difference = –0.01 in N and noT conditions and 0.0 in noA condition. In all conditions it can be asserted that the true (population) difference between the two methods is negligible, because the larger limit (defined as *Pr**[ |population difference| < limit] = 0.95) is only 0.01 (1% of the full rating scale).
