# Supplementary material for: Investigating the Role of Auditory and Tactile Modalities in Violin Quality Evaluation
Source: PLoS One. 2014 Dec 4;9(12):e112552. doi: 10.1371/journal.pone.0112552 (PMC4256376; doi:10.1371/journal.pone.0112552)
Supplement: Appendix S2 — Effect of violin on preference ratings under noA condition. (DOCX) [file pone.0112552.s002.docx]

**Appendix 2: Effect of violin on preference ratings under noA condition**

The preference ratings in the normal condition were subtracted from those in the noA condition for the 19 participants who completed the task. The overall raw effect of violin, measured as Root Mean Square (RMS) of the pairwise mean differences, is RMS = 0.26. A repeated-measures ANOVA on the difference between ratings shows a significant effect of violin, *F*(3.66,65.95) *=* 5.42*, ε =*0.75*, p <* 0.01, which means that the effect of auditory masking on preference ratings depends on the violin (*Pr^*^*[population RMS > 0.18] = 0.95). Post-hoc analyses (with Bonferroni correction) show that violin VA is significantly different from violins VC (*p* =0.03), VD (*p* = 0.01) and VE (*p* = 0.02).
